# Experiences with Medications for Addiction Treatment Among Emergency Department Patients with Opioid Use Disorder

**DOI:** 10.5811/westjem.2022.9.57821

**Published:** 2023-02-22

**Authors:** Charlotte E. Goldfine, Brittany P. Chapman, Melissa M. Taylor, Evan S. Bradley, Stephanie P. Carreiro, Rochelle K. Rosen, Kavita M. Babu, Jeffrey T. Lai

**Affiliations:** * Brigham and Women’s Hospital, Division of Medical Toxicology, Department of Emergency Medicine, Boston, Massachusetts; † University of Massachusetts Chan Medical School, Division of Medical Toxicology, Department of Emergency Medicine, Worcester, Massachusetts; ‡ Brown University School of Public Health, Department of Behavioral and Social Sciences, Providence, Rhode Island

## Abstract

**Introduction:**

Medications for addiction treatment (MAT) are the evidence-based standard of care for treatment of opioid use disorder (OUD), but stigma continues to surround their use. We conducted an exploratory study to characterize perceptions of different types of MAT among people who use drugs.

**Methods:**

We conducted this qualitative study in adults with a history of non-medical opioid use who presented to an emergency department for complications of OUD. A semi-structured interview that explored knowledge, perceptions, and attitudes toward MAT was administered, and applied thematic analysis conducted.

**Results:**

We enrolled 20 adults. All participants had prior experience with MAT. Among participants indicating a preferred treatment modality, buprenorphine was the commonly favored agent. Previous experience with prolonged withdrawal symptoms upon MAT discontinuation and the perception of “trading one drug for another” were common reasons for reluctance to engage in agonist or partial-agonist therapy. While some participants preferred treatment with naltrexone, others were unwilling to initiate antagonist therapy due to fear of precipitated withdrawal. Most participants strongly considered the aversive nature of MAT discontinuation as a barrier to initiating treatment. Participants overall viewed MAT positively, but many had strong preferences for a particular agent.

**Conclusion:**

The anticipation of withdrawal symptoms during initiation and cessation of treatment affected willingness to engage in a specific therapy. Future educational materials for people who use drugs may focus on comparisons of respective benefits and drawbacks of agonists, partial agonists, and antagonists. Emergency clinicians must be prepared to answer questions about MAT discontinuation to effectively engage patients with OUD.

## INTRODUCTION

Non-fatal opioid overdoses outnumber fatal overdoses by 20 to 1.^1^ Most individuals who receive naloxone from first responders are transported to an emergency department (ED).^2^ In 2017, more than 965,000 patients were treated in EDs after non-fatal opioid overdoses.^3^ Thus, the ED visit following an opioid overdose represents a critical opportunity for healthcare workers to offer evidence-based interventions for opioid use disorder (OUD), including medications for addiction treatment (MAT), to a high-risk and vulnerable population.^4^,^5^ Initiating MAT in the ED has been shown to improve retention in treatment for OUD at 30 days.^6^ Moreover, treatment of OUD with methadone or buprenorphine in the year following a non-fatal overdose is associated with marked reductions in all-cause and opioid-related mortality.^7^

Given the fulminant course of these patients – 5% will die within the year – opioid agonist or partial-agonist treatment from the ED should be offered to all patients who present after non-fatal overdose.^8^ Yet few people who use drugs receive MAT despite increases in availability of this treatment.^9^ Experts have postulated that key barriers are stigma, logistical issues, clinician lack of training on OUD treatment, and gaps in patients’ knowledge regarding treatment options.^10^ However, knowledge and attitudes of ED patients toward MAT have not been adequately elucidated. We sought to improve understanding of patient attitudes, misconceptions, and barriers to MAT to facilitate engagement with MAT from the ED. Specifically, we used a semi-structured interview to assess the following: 1) familiarity with MAT; 2) attitudes toward and experience with methadone, buprenorphine, naltrexone, and abstinence-based treatment; 3) experiences with withdrawal symptoms; and 4) treatment acceptability.

## METHODS

This was a qualitative study of adult patients who presented to an ED with an opioid-related chief complaint. This study was approved by the University of Massachusetts Chan Medical School Institutional Review Board, employed an exploratory qualitative design, was not hypothesis-driven, and was not pre-registered in a publicly available platform.

### Setting

Massachusetts is the most populous state in the New England region of the United States (US). Its population of 6.9 million has been disproportionately affected by the opioid epidemic, with an overdose mortality rate of 29.3 per 100,000 in 2018, fifth highest in the United States.^11^,^12^ The University of Massachusetts Memorial Medical Center is the sole tertiary-care academic referral hospital in central Massachusetts. Its ED sees an annual volume of 130,000 visits, with approximately 600 patients per year presenting for evaluation of complications of OUD. This population is at especially high risk of morbidity and mortality from drug use, representing a group in whom targeted education and harm reduction efforts might yield the greatest benefit.^8^ Study staff sought to achieve a sample that is representative of the population of people who use drugs in the region, with respect to gender, age, drugs injected, duration of drug use, and prior experiences with OUD treatment.

### Recruitment

The electronic health record ED tracking board was used to screen for individuals with an opioid-related chief complaint (eg, overdose, abscess, request for detox). Potential participants were approached once they had been deemed medically stable by their treating attending physician. A convenience sample was enrolled during the study period (March–November 2019). Eligible participants were 18 years of age or older, presented to the ED with an opioid-related chief complaint, had a history of OUD, were English-speaking, and able to provide informed consent. Individuals were excluded if they had previously participated in this study or were in police custody. Verbal informed consent was obtained.

**Population Health Research Capsule**

What do we already know about this issue?

*Medications for addiction treatment (MAT) comprise the evidence-based treatment of opioid use disorder, yet stigma and barriers to access limit their use*.

What was the research question?

*We sought to improve understanding of emergency department (ED) patient attitudes, misconceptions, and barriers to MAT*.

What was the major finding of the study?

*Participants viewed MAT positively, yet acceptance was curbed by anticipated withdrawal upon cessation*.

How does this improve population health?

*By addressing concerns around discontinuation of MAT and educating patients on its benefits, we can increase engagement in MAT among ED patients*.

### Data Collection

Study investigators administered a brief demographic survey, followed by a semi-structured interview consisting of open-ended questions regarding experiences with naloxone and opioid withdrawal, attitudes toward MAT and recovery, and familiarity with naltrexone, buprenorphine, and methadone (Appendix A and B). The semi-structured interview guide was developed by senior investigators with prior expertise in qualitative research techniques. Interviews were conducted by trained interviewers with prior experience in other qualitative, open-ended research studies with similar populations. At the conclusion of the interview, participants were compensated for their time with a $10 gift card for a local retail store. We tabulated and managed demographic data using REDCap electronic data capture tools hosted at the University of Massachusetts Chan Medical School.^13^,^14^ Semi-structured interviews were audio recorded and professionally transcribed. After 20 interviews, senior investigators met with the interviewers to discuss the data collected. Considering that the demographics of the interviewed participants matched that of people who use drugs seen in the ED and that most of the interview questions were addressed by the interviewees, we determined that the sample size was sufficient to adequately explore themes of interest.^15^

### Data Analysis

We used an applied thematic analysis and framework matrix analysis to analyze the qualitative interviews. Deductive codes were developed by study investigators (JL and BC) from key topics of interest in the interview guide. Deductive codes included attitudes and experiences with naltrexone, suboxone, and methadone. Additional inductive codes were developed during review of all transcripts to capture novel and emergent concepts brought forth by the participants. Inductive codes included participants’ discussion of their experiences of pain and withdrawal. Codes were organized as parent codes, with subcodes representing more specific themes within each category. We found that many of our top level/parent codes were deductive, but that additional subcodes were added to these categories based on participants’ concepts, such as the misuse potential of MAT. The majority of our codes were deductive, and approximately six of the 48 codes and subcodes were inductive.

Two researchers (JL and KB) independently openly coded the first two interview transcripts. The obtained codes were reviewed by the research team in aggregate and adjusted as needed (eg, codes were renamed or their definitions clarified). This resulted in a preliminary thematic coding scheme. New codes were created as needed during review of three more transcripts. Throughout this process, codes were discussed and refined until agreement between the researchers was reached. After five interviews, no further changes were made to the codes. The finalized codes were then used on the remaining transcripts, which were double coded by two study investigators (JL and KB) and reviewed and verified by two additional researchers (BC and MT). Differences in coding were resolved and the agreed-upon codes were entered into NVivo 12 Plus (QSR International, Burlington, MA) to complete the thematic analysis and generate summaries of key topic areas. We also made note of important or unique findings. Quotations illustrating relevant themes were selected for presentation.

## RESULTS

A total of 47 participants were screened for recruitment. Twenty-two were unable to be approached because they exhibited altered sensorium (11/22); eloped from the ED (3/22); study staff were unavailable to administer the interview (5/22); or other (3/22). Of the 25 individuals approached, five declined to participate because they did not feel well enough to complete the interview (two patients), had no interest in participating (one), or requested immediate discharge from the hospital (two). Twenty participants were enrolled in the study (Table 1). The sample was comprised predominantly of young, White males with prior experience with OUD treatment. The sample varied with respect to educational attainment, current employment, and housing status.

Analysis of semi-structured interviews revealed several themes, described in detail below. Additional illustrative quotations are included for each theme (Table 2).

### Experience with Opioid Use Disorder

Most participants (15) had a prior opioid overdose for which they had received naloxone. Nine participants also reported receiving opioid reversal more than once; one participant described being reversed with naloxone “too many [times] to count.” Most reported a positive perception of naloxone and were thankful to have received it. Participants possessed a high degree of functional knowledge regarding MAT. All 20 participants had prior direct personal experience with MAT, and individuals were most familiar with methadone and buprenorphine.

### Attitudes Toward and Experience with Methadone

Fourteen participants reported prior treatment with methadone. Some participants were unsure of the mechanism of methadone, and one participant mistook it for an opioid “blocker.” Participants viewed methadone positively because it ameliorated withdrawal symptoms during detox, treated pain, improved craving, and facilitated a return to normal daily activities. Participants cited the lack of a required washout period prior to starting methadone as a benefit. One participant identified boredom as a trigger for their opioid use, and thus liked the regimented nature of daily visits to the methadone clinic; the clinic they attended also offered groups and intensive outpatient treatment that helped mitigate the risk factor for return to use.

However, other participants expressed significant reservations regarding methadone. They disliked that they felt “high” from methadone and described it as “more of a substitute for drugs” compared to other treatment options. Several participants found the daily clinic visits to be inconvenient, particularly in extreme weather when they needed to “stand out there in the snow.” Others cited concerns regarding prolonged and severe withdrawal symptoms with methadone discontinuation and stigma related to methadone treatment.

### Attitudes Toward and Experience with Buprenorphine

Seventeen participants reported prior treatment with buprenorphine. Most participants viewed buprenorphine favorably; one participant called it a “wonder drug.” Participants described improvement of withdrawal symptoms, decreased pain, feeling normal/“not high,” and ameliorated cravings. One participant had used buprenorphine extended-release injection (Sublocade) and liked the convenience of the 28-day cycle.

One participant noted buprenorphine did not improve withdrawal symptoms after using heroin/fentanyl. Other participants reported adverse effects, such as withdrawal symptoms with discontinuation or missed doses, drowsiness, bad taste/smell, restlessness, nausea, and precipitated withdrawal. While only one participant reported difficulty obtaining a buprenorphine prescription, eight described purchasing illicit buprenorphine to self-treat withdrawal symptoms. Additional reported barriers included the frequency of clinic visits for prescription renewal, concern for untreated pain; desire for more structured programs or concurrent psychiatric treatment; preference for drug-free abstinence; and financial pressures to sell buprenorphine. Some participants were also concerned about the misuse and diversion potential of buprenorphine, and self-reported prior use of buprenorphine to get high or sell their prescription in exchange for other drugs.

### Attitudes Toward and Experience with Naltrexone

Fewer participants reported prior personal treatment history with naltrexone (n=6), compared to methadone (n=14) and buprenorphine (n=17). However, most knew someone who had previously been prescribed naltrexone and reported those people described a positive experience due to the inability to use opioids and improvement in cravings.

Most participants described the mechanism of naltrexone as an opioid “blocker.” Participants were largely familiar with Vivitrol by brand name, but frequently conflated naltrexone with naloxone due to the similarity of the generic names. Most participants knew naltrexone was formulated as an intramuscular injection, and six participants knew of the pill formulation. Nine participants correctly reported that the effects of injectable naltrexone last for 30 days, while one participant erroneously thought it lasted for 3–6 months.

Presented with a hypothetical scenario in which someone on long-term naltrexone treatment attempted to use opioids, some participants correctly stated that the individual would experience no euphoric effects or could possibly experience euphoria if they used a sufficiently large opioid dose. However, others incorrectly reported that this individual would experience no euphoria but experience imminent death or would experience opioid withdrawal symptoms.

Most participants with prior naltrexone treatment experience regarded it positively. One stated benefit of depot naltrexone was not having to “worry about … not taking that pill one day and then grabbing a bag instead and that being the last day I have on this Earth.” Other reported benefits included ease of use, monthly rather than daily administration, and less stigma. Additionally, several participants described that naltrexone helped with cravings. One stated, “[Vivitrol’s] a mind controller, you know. It really help[s] you stop thinkin’ about [opioids].” Some participants felt there were no side effects or dangers of taking naltrexone, while others reported that potential adverse effects include withdrawal symptoms, overdose, ability to break through the blockade, allergic reaction or rash, depression, injection site soreness, and nausea.

While most participants reported that methadone and buprenorphine were solely for the treatment of OUD, some participants believed that naltrexone was effective for substances beyond opioids (eg, cocaine, “all drugs”). Most participants were familiar with naltrexone also being used for alcohol use disorder. Five participants perceived no barriers to receiving naltrexone. Three participants were concerned about being unable to tolerate withdrawal symptoms prior to naltrexone initiation. Additional barriers included a preference for abstinence-based treatment, difficulty with transportation, risk of relapse or overdose prior to the next dose, desire for the ability to get high, and perceived inability to treat pain.

Participants reported receiving information about naltrexone from OUD treatment programs, from other people who use drugs with prior naltrexone treatment experience, pamphlets, physician, and jail. Seven participants were interested in receiving additional information about naltrexone while eight were not. Participants were interested in learning how and why naltrexone works; adverse effects and toxicity; where and how to access it; positive and negative effects; and whether it had euphoric effects.

### Attitudes Toward and Experience with Abstinence-based Treatment

Participants varied in their definition of sobriety, with some defining their goal in recovery as drug-free abstinence, whereas others viewed MAT as a vital part of their recovery. Some participants had experience with abstinence-based treatment; however, most participants reported this usually resulted in return to drug use. The most common reasons for preferring abstinence-based recovery were stigma associated with MAT use and concern that MAT was substituting one drug for another. Additionally, some participants reported involvement with abstinence-based groups as a reason for not wanting MAT, perceiving that these groups equated MAT use with not being sober. Among participants who preferred drug-free abstinence, most acknowledged that abstinence-based sobriety was difficult to achieve from the outset and viewed MAT as a bridge to this long-term goal.

### Experience with Withdrawal Symptoms

All but one participant reported previously experiencing symptoms of opioid withdrawal. While many participants felt they could tolerate withdrawal symptoms for a short duration, most felt an extended withdrawal period was unacceptable. While physical symptoms of opioid withdrawal were common, the most intolerable withdrawal symptoms were neuropsychiatric: insomnia, anxiety, lack of autonomy/feeling controlled by withdrawal symptoms, and hopelessness. Some participants recounted such a strong emotional response that even the thought of withdrawal made them anxious.

### Treatment Acceptability

While MAT was generally accepted, several individuals cited the misuse potential of methadone and buprenorphine as reasons for wanting to avoid these therapies. Most participants expressed the importance of having a plan in place to taper off agonist treatment prior to initiation, due to previously experiencing prolonged withdrawal. Many participants were accepting of partial agonist medications (buprenorphine), with seven participants describing it as their preferred treatment modality. Five participants reported they would prefer naltrexone, while others cited precipitated withdrawal symptoms as their main reason for avoiding this medication. Only one participant reported methadone as their preferred medication. There were two participants who reported they would opt for an abstinence-based recovery. Participants also expressed interest in more mental health treatment combined with MAT.

Participants were eager for more information about treatment options, preferring to learn about MAT through discussions or reading materials. Although most participants wanted these conversations to be with a clinician, a few participants preferred to learn from people who use drugs who had personal experience with the treatment options. One participant suggested the information should be easily understood, while another participant preferred to have access to the primary literature.

## DISCUSSION

In our sample of 20 ED patients with OUD, all participants had prior experience with MAT; 85% with buprenorphine, 70% with methadone, and 30% with naltrexone. Overall, participants viewed MAT positively. Many participants held strong preferences for a specific agent but differed in the reasons for these preferences. Participants often reported that their own prior experiences, or those of people they knew, influenced their attitudes toward a particular form of MAT.

In a previous qualitative study of people who use drugs in rural New Mexico, participants had more experience with buprenorphine than methadone, felt that treatment with MAT improved withdrawal symptoms and quality of life, preferred buprenorphine to methadone, and cited dislike for being dependent on MAT due to stigma and the perception of substituting one drug for another.^16^ It is noteworthy that individuals in environments as disparate as rural New Mexico and urban New England share such similar perspectives regarding MAT. Our results underscore the importance of combating the stigma associated with OUD, addressing common fears surrounding MAT and opioid withdrawal, and understanding individual definitions of sobriety.

Our results should inform discussions with people who use drugs and refine OUD treatment programs. Importantly, the current standard of care for treating opioid withdrawal consists primarily of medications that ameliorate its physical symptoms but do little to mitigate the psychological symptoms that were reported to be far more unpleasant. Additionally, it is imperative to note that when people who use drugs are engaging in OUD treatment, many are already thinking ahead to when they may be discontinuing MAT and considering potential withdrawal effects as a significant factor in evaluating the suitability of a particular form of MAT. Therefore, engagement in initiating MAT among ED patients may be improved by addressing not only the current withdrawal symptoms and short-term benefits but also long-term concerns such as potential withdrawal symptoms when discontinuing MAT. This knowledge should be leveraged in the initial discussions of treatment options, to help inform people who use drugs of the advantages and disadvantages of each, and to empower them to select the option most suitable for their individual circumstances. Lastly, naltrexone may be an acceptable treatment modality for individuals who wish to pursue drug-free recovery.

## LIMITATIONS

The main limitation of the present study is the lack of diversity among the study participants, who were mostly young, White males. The population is representative of the typical sample of people who use drugs in our region, and we did not find a difference in characteristics between approached vs enrolled participants. Because this was a convenience sample, there is a possibility for selection bias in which participants more comfortable with discussing their OUD agreed to participate in the qualitative interview.

There were also several limitations inherent in this qualitative research project. All study staff were trained in qualitative interview techniques; however, interviews were conducted by three different interviewers. Consequently, there is potential for variation in the way questions were asked, as is to be expected in a semi-structured qualitative interview. Data was analyzed by the qualitative interviewers; coding credibility and reliability were addressed by having two independent reviewers code the data, which was then reviewed and verified by two additional reviewers, each of whom individually reviewed the codes and entered the data. Thematic analysis was written using coding summaries and notes from the qualitative data. Themes were written by JL and reviewed by all analysts at team meetings to ensure agreement about the interpretation and representation of this data.

## CONCLUSION

Overall, participants had a positive view of medications for addiction treatment but tended to have strong preferences for a particular agent, based upon previous personal experience or anecdotes from people who use drugs that they knew. Willingness to engage in a specific therapy was affected by the perceived likelihood of experiencing withdrawal symptoms and their anticipated severity, both during treatment initiation and cessation. Future outreach efforts should specifically elicit an individual’s conceptualization of sobriety and address the relative benefits and drawbacks of agonists, partial agonists, and antagonists within that framework.

## Supplementary Information





## Figures and Tables

**Table 1:** Participant demographics.

Age, years	
Mean	38.35
Standard deviation (population)	10.52
Median	32.5
Sex, n (%)	
Male	15 (75)
Female	5 (25)
Race, n (%)	
White	14 (70)
Black	3 (15)
Multiracial	2 (10)
Other	1 (5)
Ethnicity, n (%)	
Hispanic or Latino	4 (20)
Non-Hispanic or Latino	16 (80)
Current living situation, n (%)	
House	4 (20)
Apartment	7 (35)
SUD treatment facility/sober living	2 (10)
Homeless	7 (35)
Highest degree/level of school completed, n (%)	
Some high school, no diploma	7 (35)
High school graduate, diploma or equivalent	6 (30)
Trade, technical, or vocational training	2 (10)
Some college credit, no degree	3 (15)
Associates degree	2 (10)

*SUD*, substance use disorder.

**Table 2:** Illustrative quotations.

Theme	Quote
Experience with opioid use disorder	“For the last couple of years, … I have not wanted to get high, like shoot dope… I’m 50 … years old. My life sucks. Drugs… have done a number on me… Drugs will take and steal … everything out of your life - until you have no life… I was the … postman. I was a homeowner. I loved my wife. [We had] a beautiful daughter… On my way to work, car accident… This is the late ‘90s. The doctor was like, ‘…there’s something better out there [than Percocet]. It’s Oxycontin.’ And now here I am in 2020— still struggling with demons.” “I mean just not getting high for a month, it can change a whole lot of stuff, and getting high one time in a year and a half could change a whole lot of stuff.” “No matter what, I’m an addict for life. I admit that. I have an addict mentality. It’s gonna be with me… for the rest of my life. ’Til I’m 100 years old… You know what the difference is? Is whether I pick up something or I don’t. You know what I mean?”
Attitudes toward and experience with naltrexone	“I have three of my friends right now that are on Vivitrol … they’re telling me every day, “Get Vivitrol. Get Vivitrol.” … You don’t have to think—wake up the next day and say, “Should I take a Sub or should I get high?” You’re already wakin’ up because you know you can’t get high.” “[I want to go from Suboxone to Vivitrol] to not have to … worry about … not taking that pill one day and then grabbing a bag instead and that being the last day I have on this Earth—” “And if they know they can’t get high, they don’t use, and their life gets better, and slowly, they see the improvement in a period of 28 days.” “The Vivitrol gets a lot more respect than like people that are on methadone or Suboxone —”
Attitudes toward and experience with methadone	“I don’t like methadone … only because it’s … more of a substitute for drugs.” “I like it because … it actually gets ya high. I don’t like it because it’s the worst come down in the world… Honestly, I think methadone is harder to come off of than heroin.” “Cause there’s no detox… You can just go in there and take methadone, and you’re all set.”
Attitudes toward and experience with buprenorphine	“[Suboxone] makes me feel like I didn’t ever do heroin. I’m not sick anymore. I’m perfectly normal.” “It’s a wonder drug. It really is. It’s great. It’s never failed me.”
Attitudes toward and experience with abstinence-based treatment	“I prefer not to be on any type of maintenance or anything [because] I have mental health issues, and it gives me a better baseline to see where I’m at. Plus, I honestly don’t consider that being clean… if I still have to go take an opiate every single day.” “but there is a certain amount of weakness, especially, I think, in men, that comes when one might have to use another drug in order to keep them off of another drug.”
Experience with withdrawal symptoms	“I don’t feel autonomous. I don’t feel in control of myself. I feel like the withdrawals are controlling everything I’m doing.” “Hooked, when you stop, you see how you feel. You’d be calling your friend, or callin’ your mother, callin’ someone so you can get some money so you can buy some [heroin] so you’re not sick.”
