# Supplementary material for: Experiences with Medications for Addiction Treatment Among Emergency Department Patients with Opioid Use Disorder
Source: West J Emerg Med. 2023 Feb 22;24(2):236–42. doi: 10.5811/westjem.2022.9.57821 (PMC10047725; doi:10.5811/westjem.2022.9.57821)
Supplement: Supplementary file 1 [file wjem-24-236-s001.docx]

**Qualitative Agenda for Alkermes Naltrexone Grant**

**Semi-Structured Interview**

**May 30, 2019**

*The intent of these interviews is to understand participants’ knowledge about, and experiences with, naltrexone therapy for opioid use disorder. We want to understand their attitudes and beliefs, as well as practical barriers to participation in naltrexone treatment. We also want to know their preferred avenues for receiving educational information regarding naltrexone therapy.*

*This agenda is intended to guide facilitators through the key content areas of data collection for this project, ensuring that the same content is discussed in each interview. While the agenda is used to guide the discussion, it is not a rigid script that will be adhered to verbatim. This ensures that the facilitators gather data on the same topics in each interview, while also allowing them the flexibility to adapt and clarify questions to suit the needs of each participant. Similarly, questions need not be asked in this particular order. Rather, the facilitators will adapt the conversation as needed according to the narrative within each interview, pursuing both the a priori* *research topics as well as any emergent relevant themes that evolve from the discussion.*

**A. Welcome**

***Intent*:** *To welcome the participant and explain the purpose of the interview. We will introduce the facilitator and notetaker (if applicable) and explain how the interview fits into the larger research project.*

1. Greet the participant and introduce yourself.
2. *Explain the study:*
   1. The purpose of our study is to guide treatment and learn how our community perceives naltrexone. This interview will allow us to better help others get into treatment.
   2. We’d like to interview you to learn more about your knowledge and experiences with naltrexone, as well as any barriers (e.g., insurance, cost, transportation) that you anticipate to successful participation in treatment with naltrexone.
   3. We will also ask about what forms of naltrexone educational materials that you would find most useful and engaging (e.g., pamphlets, videos, social media posts).
   4. To thank you for your time and participation, we have a $10 gift card to a local business where you can get groceries or other household items.
   5. You are the expert here; we want to learn your perspective about naltrexone.
3. The goal is to finish this conversation in about 30 minutes. In order to cover all of the topics, we may choose to shorten a discussion on one subject in order to move along to the next.
4. *Important:* You do not have to answer any question that you do not want to. You can simply say “I’d like to pass on that” and we’ll move on.
5. We are recording the interview and will make a transcript and notes from it. The transcript and notes will not contain identifying information; your data will only be labeled with a randomly generated study ID number. We will destroy the audio recording after the transcript is finalized. The deidentified transcript and notes will be shared with other researchers who are helping with the analysis. No other recording is allowed; I am going to ask you to turn off your phone.
6. Do you have any questions before we begin?
7. Do you agree to participate in this study and have this interview audio recorded?
8. **Turn on** the recorder(s).
9. Start the interview by stating “Today is ___ / ___ / ___ and this is going to be an Alkermes interview for ID# ______.”

**B. Previous experience with medication-assisted treatment**

***Intent:*** *The intent of this section is to understand the participant’s prior experiences with and perceptions of treatment options for opioid use disorder.*

***Framing statement:*** I want to understand what your experience has been with treatment for opioid use disorder.

1. In the past **6 months**, have you **tried** to get treatment for your drug use?
   1. *(If yes)*
      - How?
      - What kind of treatment?
      - Were you able to enter/start treatment?
   2. *(If no)* Have you **ever** tried to get treatment for your drug use?
      - *(If no) Skip to #4*
2. Were there any difficulties in getting into these programs?
   1. *(If yes)* What difficulties did you encounter? *Probes:* Insurance? Transportation? Family obligations? Work obligations?
      - Were you able to overcome these barriers?
        - *(If yes)* How?
      - Did you seek help from others in overcoming these barriers?
3. To confirm, in the past **6 months**, you have engaged in [LIST]. Did you feel like these were helpful in your treatment?
   1. *(If no)*
      - Why?
      - What aspects were unhelpful or counterproductive? Why?
      - What can these programs do differently so that they are helpful?
   2. *(If yes)*
      - Why?
      - Which was the most helpful? Why?
      - Is there anything you’d like these programs to do differently?
      - Were there any aspects of these programs that you found to be unhelpful or counterproductive? Why?
4. Have you **ever** received naloxone (Narcan^(R)^) for an overdose before?
   1. *(If yes)* When?
5. Have **ever** you taken/received naltrexone or Vivitrol^(R)^?
   1. *(If yes)*
      - Can you tell me about your experience **starting** this medication? *Probes:* Where/who did you first hear about it from? Did you have to wait a certain period of time before you could start the medication? Did you experience any unpleasant symptoms before/after? Did you take pills or receive shots?
      - When was the last time you took it/received a shot?
        - *(If no longer receiving naltrexone treatment)* Why did you stop?
      - How long were/have you been taking naltrexone?
      - Can you tell me about your **overall experience** with this medication? *Probes:* Likes? Dislikes?
6. Have you taken methadone, naltrexone (Vivitrol^(R)^), or buprenorphine (Suboxone^(R)^) in the **last month**?
   1. *(If yes)*
      - Did you find it to be helpful?
        - *(If yes)* In what ways?
        - *(If no)* Why not?
      - Was it/were they prescribed by a doctor or treatment program?
        - *(If no)* How did you obtain it/them?
      - When was the last time you took it/them?
        - *(If recently stopped)*
          - How long had you been taking it for?
          - Why did you stop?

**C. Knowledge, beliefs, and attitudes regarding naltrexone**

***Intent:*** *The intent of these questions is to understand what the participant knows about naltrexone, as well as elucidate attitudes and beliefs of participants and their acquaintances regarding naltrexone.*

1. Can you tell me what you know about naltrexone? Can you tell me what you know about Vivitrol^(R)^?
   1. *(If the participant has never heard of naltrexone or Vivitrol^(R)^, give them following information and skip #2)* Naltrexone is a medication that can be used for treatment of alcohol use disorder or opioid use disorder. It is available both as a pill and a shot. It differs from other treatment options, in that methadone and Suboxone^(R)^ both bind to the opioid receptor and produce some activity there, while naltrexone binds to the opioid receptor and is a pure blocker.
2. How is naltrexone different from other medications that are used to treat opioid use disorder?
   1. *Probes:* Can you get high from naltrexone itself? Are there side effects of naltrexone? Are there dangers to naltrexone? Can you use opioids while you are on naltrexone?
3. Without naming names, do you know anyone who has been on naltrexone in the **past**, or **currently**?
   1. *(If yes)* Did they say anything about their experiences? Likes? Dislikes?
      - *(If yes)* What did they say?
4. Would you want to learn more about naltrexone?
   1. *(If yes)*
      - What would you want to know?
      - What methods would you find most helpful to learn more about naltrexone? *Probes:* Pamphlet? Website? Phone app? Video? Talking to a clinician?
   2. *(If no)* Are there any particular reasons as to why?
5. If all other aspects of a treatment program were the same, which of the commonly used medications for opioid use disorder (e.g., methadone, buprenorphine (Suboxone^(R)^), naltrexone (Vivitrol^(R)^)) would you be most likely to accept as part of your treatment?
   1. Why?

**D. Readiness to initiate naltrexone treatment**

***Intent:*** *Here we want to understand the participant’s readiness to begin oral naltrexone treatment as a bridge to intramuscular naltrexone treatment, their willingness to tolerate opioid withdrawal symptoms to accelerate initiation of oral or intramuscular naltrexone, as well as any barriers to naltrexone treatment that the participant may foresee.*

***Framing statement:*** We would like to find out how people engage with opioid treatment programs, and in particular naltrexone treatment programs. I would like to ask you some questions about any difficulties you might have encountered in the past, as well as the degree of withdrawal symptoms that you would be willing to tolerate as part of the process of initiating treatment.

1. Do you think there are there any circumstances that would hinder your participation in a naltrexone treatment program?
   1. *(If yes)* What are they? Do you think they can be overcome?
2. ***Framing statement:*** Opioid withdrawal can be uncomfortable. One of the concerns, historically, regarding naltrexone is that it can produce withdrawal symptoms if it is started too early, while there is still an opioid in your system. However, we are trying to find ways to get people plugged into treatment directly from the emergency department. If it meant that you could be started on a treatment medication at the time you are being evaluated for an overdose, would you be willing to tolerate withdrawal symptoms if it meant you could be started on medications right away?
   1. *(If yes)* Why?
      - What degree of withdrawal would you be willing to tolerate in order to be initiated on treatment?
   2. *(If no)* Why not?
3. What withdrawal symptoms are most bothersome to you?
4. Would you be willing to engage in a trial of oral naltrexone after you are seen in the emergency department, with a plan to follow up in clinic for a dose of intramuscular (long-acting) naltrexone?

**E. Participant initiated topics**

1. Is there anything else that you want to share, or you think is important for us to know?
2. *Stop the recordings.*

**F. Demographics form**

Ask the participant to complete the brief demographics survey on the tablet computer or paper form. Allow the participant to answer questions on their own and provide guidance as needed. Collect the tablet or paper form once completed.

**G. Conclusion**

1. Thank you for your participation in this research. We greatly appreciate your time and input.
2. Distribute compensation.

**H. Debriefing/reflection**

***Intent:*** *The intent of this section is to write a general reflection about the interview that includes thoughts about the subjects’ attitudes, reactions, and any other potentially pertinent information. This should be done immediately after the interview, once you have left the subjects’ area.*

Study Staff Reflection:
